# Stress-Induced Allodynia – Evidence of Increased Pain Sensitivity in Healthy Humans and Patients with Chronic Pain after Experimentally Induced Psychosocial Stress

**DOI:** 10.1371/journal.pone.0069460

**Published:** 2013-08-07

**Authors:** Benjamin Crettaz, Martin Marziniak, Peter Willeke, Peter Young, Dirk Hellhammer, Astrid Stumpf, Markus Burgmer

**Affiliations:** 1 Department of Psychosomatics and Psychotherapy, University Hospital Münster, Münster, Germany; 2 Department of Neurology and Department of Inflammatory Disorders of the Nervous System and Neurooncology, University Hospital Münster, Münster, Germany; 3 Department of Medicine D, Section of Rheumatology, University Hospital Münster, Münster, Germany; 4 Department of Sleep Medicine and Neuromuscular Disorders, University Hospital Münster, Münster, Germany; 5 Department of Psychology, Trier University, Trier, Germany; Charité University Medicine Berlin, Germany

## Abstract

**Background:**

Experimental stress has been shown to have analgesic as well as allodynic effect in animals. Despite the obvious negative influence of stress in clinical pain conditions, stress-induced alteration of pain sensitivity has not been tested in humans so far. Therefore, we tested changes of pain sensitivity using an experimental stressor in ten female healthy subjects and 13 female patients with fibromyalgia.

**Methods:**

Multiple sensory aspects of pain were evaluated in all participants with the help of the quantitative sensory testing protocol before (60 min) and after (10 and 90 min) inducing psychological stress with a standardized psychosocial stress test (“Trier Social Stress Test”).

**Results:**

Both healthy subjects and patients with fibromyalgia showed stress-induced enhancement of pain sensitivity in response to thermal stimuli. However, only patients showed increased sensitivity in response to pressure pain.

**Conclusions:**

Our results provide evidence for stress-induced allodynia/hyperalgesia in humans for the first time and suggest differential underlying mechanisms determining response to stressors in healthy subjects and patients suffering from chronic pain. Possible mechanisms of the interplay of stress and mediating factors (e.g. cytokines, cortisol) on pain sensitivity are mentioned. Future studies should help understand better how stress impacts on chronic pain conditions.

## Introduction

Stress is defined as the physiological reaction to real or potential life-threatening conditions [1] and is accompanied by changes in the associated neural, endocrinological and immunological systems [2]. Studies on animals have shown that experimentally induced short-term stress has not only an analgesic effect of [3], [4] but also elicits reactions of hyperalgesia and allodynia [5]–[9]. The differences in pain sensitivity seem to be influenced by the strength of the stressor and the physiological state of the animals‘ stress-system [6], [7]. Situations in need of a fight-or-flight-reaction seem to favor hyperalgesia while analgesia dominates in situations in which such an active reaction is impossible [6]. Chronic experimental stress [5], [8] and prior pain experience [7] favor stress-induced hyperalgesia/allodynia [10]. Although these animal studies suggest that stress may enhance the pain processing under certain conditions, no study has tested the concept of stress-induced hyperalgesia/allodynia in humans so far.

In humans, the onset and course of clinical pain conditions such as headache, migraine and fibromyalgia are influenced by stress [11]–[14] and are associated with changes in the hypothalamic-pituitary-adrenal axis, the core pathway of the human stress system [15]–[17]. Newer studies in animals and humans exposed to experimental stress suggest a causal relationship of cortisol levels to changes in pain sensitivity [18]–[20].

The present study describes changes pain thresholds occurring in healthy subjects under conditions of experimental stress. Because of the obvious impact of stress in clinical pain conditions, a sample of patients suffering from fibromyalgia was also investigated. The findings on the two groups were not compared due to the explorative character of our study. In line with the reports in literature, we expected to observe stress-induced analgesia in healthy subjects and stress-induced hyperalgesia/allodynia in the group of fibromyalgia patients.

## Methods

### Ethics statement

The study was conducted in accordance with the Declaration of Helsinki and approved by the local Ethics Committee of the Medical Faculty of the University Muenster, Germany.

### Participants

A total of 10 healthy female subjects and 13 patients with fibromyalgia (FMS) were recruited for the study through the University Hospital or by advertisements in local newspapers. Healthy subjects were free of any pain syndrome or current axis-I mental disorder according to the Structured Clinical Interview for DSM-IV [21]. Three of the healthy subjects regularly smoked cigarettes (4 –10) per day. On average, they drank about 2.1 cups of coffee per day and none of them were on medication. All of them discontinued nicotine and caffeine consumption 24 hours before the study.

Patients with FMS met the American College of Rheumatology criteria for FMS, had pain as their major complaint, and were free of any current axis-I psychiatric diagnosis. An extensive review of the patient charts was done by a registered rheumatologist (PW) to exclude other origins of pain, e.g. rheumatic or endocrine diseases. Three of the patients smoked cigarettes (3–20/day) and drank about 3.5 cups of coffee per day. All patients stopped nicotine and caffeine consumption 24 hours before the study. Four patients took pain medication on a regular basis (ibuprofen, citaloprame), one patient was on thyroxine substitution. Patients discontinued their medication 2 weeks prior to the study. All participants gave informed consent in writing.

### Psychometrics

One hour prior to the start of the study, the extent of affective burden during the last week was assessed in each participant with the German versions of the Beck Depression Inventory [22] and the Spielberger State-Trait Anxiety Inventory [23]. Chronic (previous month) and childhood stress exposure were assessed with the Trier Inventory of Chronic Stress [24] and the Childhood Trauma Questionnaire [25], respectively.

Furthermore, participants were asked to rate their anxiety and clinical pain (fibromyalgia only) at the time of the study on a visual analogue scale (VAS) ranging from VAS 0  =  no anxiety/no pain to VAS 10  =  unbearable anxiety/worst pain imaginable, one hour before, immediately on and 90 minutes after being exposed to the experimental stressor.

### Experimental procedure

#### Induction of experimental acute psychosocial stress

The *Trier Social Stress Test (TSST)* is a standardized and reliable psychosocial stress task [26]. The TSST consists of a preparation phase (10 minutes) followed by an activity phase of delivering a speech in front of a trained audience (5 minutes) and performing an arithmetic task (5 minutes).

Prior to stress induction, a 2-channel electrocardiograph was connected to each participant to determine stress-induced changes in heart rate and sympathovagal balance, calculated as low-frequency band power/high frequency band power*10). Both variables were recorded continuously and analyzed as blocks of 4 minutes’ duration, 10 minutes before, during (+8 min), as well as 10 and 30 minutes after exposure to the stressor.

#### Evaluation of pain thresholds

The quantitative sensory testing (QST) protocol of the German Research Network on Neuropathic Pain is a standardized method to evaluate the somatosensory phenotype of pain [27]. It assesses sensory aspects of cutaneous as well as deep pain like allodynia or hyperalgesia. We determined thermal (cold and warm) detection (CDT, WDT) and pain thresholds (CPT, HPT), mechanical pain detection thresholds to pinprick stimuli (MPT), blunt pressure pain thresholds (PPT) and pain summation (wind-up ratio, WUR) at the medial forearm of each subject.

Thermal sensation tests were performed using TSA 2001-II (Medoc, Israel), mechanical tests with modified von Frey filaments (Optihair-Set, Marstock Nervtest, Germany), and blunt pressure testing with a pressure gauge device (FDN200, Wagner Instruments, USA). The mean threshold temperature of CDT, WDT, CPT, and HPT was determined from three consecutive measurements with ramped stimuli (1 C°/s). MPT was evaluated as the geometric mean of five series of ascending and descending pressure forces with different weighted pinprick stimuli (8–512 mN). WUR was determined as the ratio of the mean rating of five series of 10 repetitive pinprick stimuli (1 Hz, 256 mN) divided by the mean rating of the five single stimuli. PPT was determined with three series of ascending stimulus intensities, each applied as a slowly increasing ramp of 50 kPa/s.

**Figure 1:**
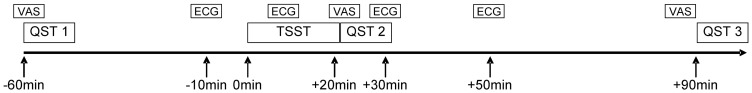
Experimental procedure. QST  =  Quantitative Sensory Testing, VAS  =  Subjects’ rating of anxiety (healthy controls and FMS) and clinical pain (FMS only) on a visual analogue scale, ECG  =  Recording of stress-induced changes of the heart rate and sympathovagal balance, TSST  =  Trier Social Stress Test.

Each QST testing was performed three times (60 minutes before the TSST, immediately thereafter and 90 minutes after the TSST) in accordance with the standardized protocol [28] at the same area of the forearm in each participant (see figure 1).

#### Data analysis

Both groups of participants were handled as independent groups and no direct group comparison was performed due to the explorative character of our study and the limited number of participants.

ANOVA with repetition was computed to analyse all pain and QST variables, variables of autonomic activation (heart rate, sympathovagal balance) and the participants’ report of anxiety or clinical pain. For both groups of subjects, the impact of the main effect ‘time’ was tested separately to determine time-dependent effects without any further group comparison. According to the recommendation of the protocol [28], data CDT, HDT, MPT, WUR and PPT were log-transformed before data analysis. Due to the possible violation of data sphericity in studies with low numbers of subjects, a Greenhouse-Geiser correction was included. Results report the F- and p-scores and the effect sizes (η^2^). For all significant results of QST variables, post-hoc t-tests were performed to determine the major time point (QST 1–3) of change.

Statistical analyses were performed with SPSS 17 (SPSS Inc.; Chicago, IL, USA) with a significance threshold of p<0.05.

## Results

### Subjects and psychometrics

Patients with fibromyalgia were older than healthy controls (mean  = 49.85 years versus 27.70 years, p<0.001). Patients scores in depression and anxiety were higher in the week prior to the study (depression, 16.25 versus 6.13, p = .013; anxiety, 48.64 versus 38.14, p = .014) and they reported more childhood trauma (63.45 versus 39.88, p = .018). There was no significant difference in the amount of chronic stress between the two groups of subjects in the preceding month, but patients with fibromyalgia showed a trend towards a greater exposure to chronic stress (20.00 versus 14.38, p = .17, see table 1).

**Table 1:** Demographic and clinical characteristics of the participants.

	HC (n = 10) mean ± sd	FMS (n = 13) mean ± sd	T-score	p-score
Age (yrs)	27.70±5.58	49.85±10.55	−6.00	**<.001**
Tender points	na	14.42±2.58		
Myalgic score	na	30.50±8.74		
Duration of disease (yrs)	na	4.69±3.9		
BDI	6.13±3.64	16.27+9.79	−2.78	**.013**
STAI (trait dimension)	38.14+7.54	48.64+8.04	−2.76	**.014**
CTQ	39.88±10.15	63.45±23.90	−2.61	**.018**
TICS	14.38±7.42	20.00±9.02	−1.44	.17

BDI  =  Beck Depression Inventory, STAI  =  Spielberger State-Trait Anxiety Inventory, CTQ  =  Childhood Trauma Questionnaire, TICS  =  Trierer Inventory of Chronic stress.

### Stress-related variables

Both groups of participants reported a medium level of anxiety prior to stress-induction, which decreased after the TSST was completed (anxiety on VAS: p = .01 for HC; p = .04 for FMS). Both groups showed an increased heart rate during the TSST, but this was significant only in FMS patients (p<.001). Sympathovagal balance did not change significantly after stress induction in either group of subjects, although a trend towards increased sympathovagal balance was detectable in patients with FMS (p = .06). Furthermore, increased clinical pain after stress-induction was detected in patients with FMS (clinical pain on VAS:p = .052, see table 2).

**Table 2:** Emotional and cardiac effects of the experimental stressor (TSST).

	HC (n = 10)	FMS (n = 13)
HR_T1	75.56±3.29	74.62±8.14
HR_T2	86.56±17.02	96.15±18.47
HR_T3	78.33±15.84	74.62±9.47
HR_T4	76.78±16.4	73.69±9.62
GLM	F = 2.12; p = .13	F = 18.15; p<**.001**
SVB_T1	13.89±4.81	20.31±8.25
SVB_T2	19.56±10.76	28.69±16.56
SVB_T3	15.00±2.74	23.31±9.56
SVB_T4	14.44±4.72	19.54±8.00
GLM	F = 2.81; p = .12	F = 3.72; p = .06
VAS_anxiety T1	25.67±19.95	41.77±28.57
VAS_anxiety T2	15.33±18.93	47.62±25.17
VAS_anxiety T3	4.33±5.92	26.92±20.92
GLM	F = 6.27; p = **.01**	F = 4.19; p = .**03**
VAS_clinical pain T1	N.a.	34.23±20.31
VAS_clinical pain T2	N.a.	45.54±22.94
VAS_clinical pain T3	N.a.	42.15±25.51
GLM		F = 3.35; p = .052

HR  =  heart rate, SVB  =  sympathovagal balance, VAS  =  visual analogue scale.

### Pain thresholds (QST)

Before stress induction, patients with FMS showed increased pain sensitivity to thermal, mechanical and pressure pain in comparison to healthy subjects (CPT, T = −2.85, p = .01; HPT, T = 2.97, p = .007; MPT, T = 3.25, p = .004; PPT, T = 4.45, p<.001). There were no changes of thermal detection thresholds or the wind-up ration (CDT: T = 1.54, p = .14; WDT, T = −1.10, p = .29; WUR: T = 0.25, p = −.07).

In *healthy controls,* the HPT revealed stress-induced allodynia/hyperalgesia with decreased levels of temperature inducing pain reactions after induction of stress (44.2 C° versus 42.2 C°, p = .057, η^2^ = .32). Changes between the first and third threshold 90 minutes after stress induction (post-hoc t-test p = .007) were mainly accounted for this significant effect. A trend (p = .08) towards allodynia/hyperalgesia was detectable with respect to CPT. No stress-induced changes in CDT, WDT, MPT, WUR or PPT could be detected.

In *patients with FMS,* stress-induced allodynia/hyperalgesia of thermal pain thresholds could be detected. After exposure to the stressor, less cold stimulation was needed to elicit pain (CPT, 18.7 C° versus 21.7 C°, p = .007, η^2^ = .43) and this difference was mainly caused by the increase directly after the stress-induction (post-hoc t-test p = .006). Patients showed lowered HPT of about 2 C° after induction of stress but with only a trend to significance (p = .06). However, in contrast to healthy controls, PPT decreased after exposure to the stressor (p = .004, η^2^ = .42) and was based upon decreased thresholds recorded immediately after administration of the stressor (post-hoc t-test p = .02).

**Figure 2:**
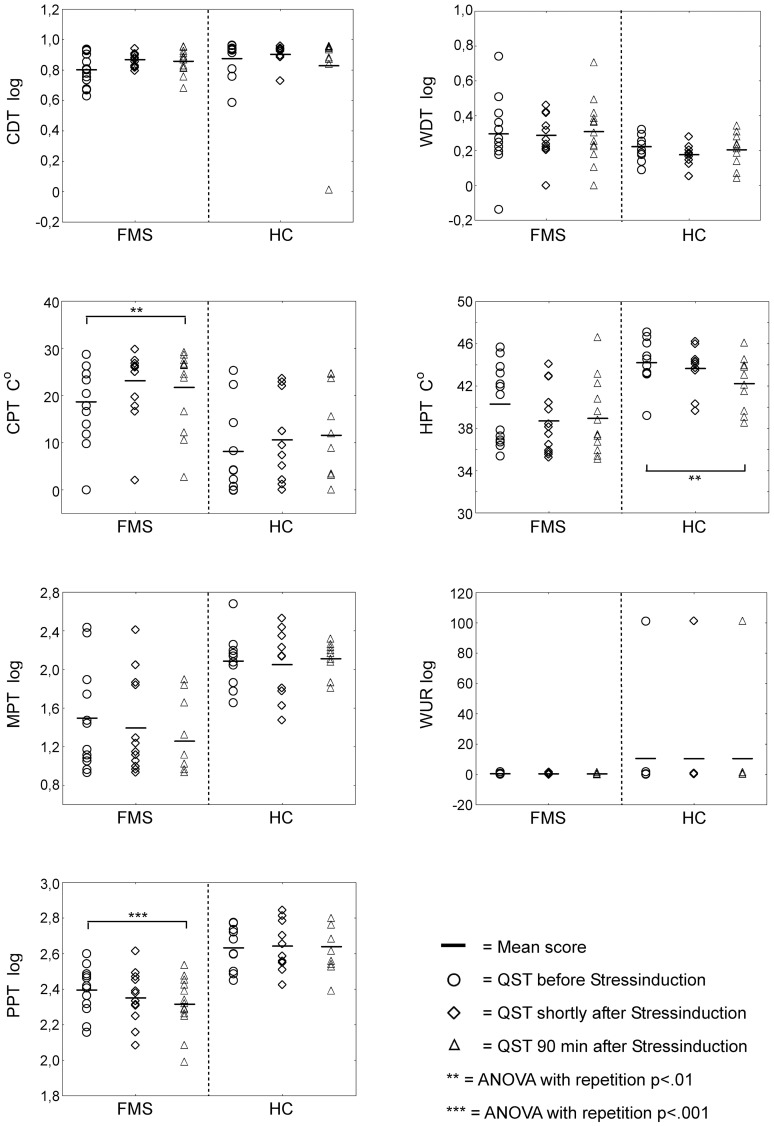
Changes of sensory pain thresholds (QST) during experimentally induced stress in healthy subjects (HC) and patients with fibromyalgia syndrome (FMS). CDT  =  cold detection threshold, WDT  =  warm detection threshold, CPT  =  cold pain threshold, HPT  =  heat pain threshold, MPT  =  mechanical pain threshold, WUR  =  wind-up ratio, PPT  =  pressure pain threshold, HC  =  healthy controls, FMS  =  fibromyalgia patients.

As in healthy controls, no changes in CDT, WDT, MPT or WUR could be detected in patients with FMS (see figure 2 and table 3).

**Table 3:** Changes of QST dimensions under stress.

	HC (n = 10)	FMS (n = 13)
CDT_T1 (C°)*	0.88±.12	0.80±.11
CDT_T2 (C°)*	0.90±.07	0.89±.08
CDT_T3 (C°)*	0.83±.29	0.86±.08
GLM	F = .61; p = .49	F = 2.79; p = .09
WDT_T1 (C°)*	0.22±.08	0.30±.20
WDT_T2 (C^o^)*	0.18±.06	0.29±.13
WDT_T3 (C^o^)*	0.20±.10	0.31±.18
GLM	F = 1.29; p = .30	F = .20; p = .81
CPT_T1 (C^o^)	8.14±9.38	18.66±8.30
CPT_T2 (C^o^)	10.60±9.25	23.13±7.63
CPT_T3 (C^o^)	11.56±10.09	21.71±8.46
GLM	F = 3.21; p = .08	F = 8.93; p = **.003**
HPT_T1 (C^o^)	44.21±2.28	40.29±3.65
HPT_T2 (C^o^)	43.66±2.14	38.70±3.09
HPT_T3 (C^o^)	42.23±2.52	38.94±3.41
GLM	F = 4.19; p = **.05**	F = 3.42; p = .06
MPT_T1 (mN)*	2.08±.29	1.50±.51
MPT_T2 (mN)*	2.05±.36	1.39±.48
MPT_T3 (mN)*	2.11±.16	1.26±.37
GLM	F = .20; p = .74	F = 2.39; p = .13
WUR_T1*	0.47±.52	0.48±.49
WUR _T2*	0.32±.23	0.45±.42
WUR _T3*	0.43±.45	0.39±.37
GLM	F = 1.58; p = .24	F = 1.65; p = .21
PPT_T1 (kPa)*	2.63±.12	2.40±.13
PPT_T2 (kPa)*	2.64±.14	2.35±.14
PPT_T3 (kPa)*	2.64±.13	2.32±.15
GLM	F = .13; p = .83	F = 8.70; p = **.004**

QST  =  Quantitative Sensory Testing, GLM  =  general linear model, CDT  =  cold detection threshold, WDT  =  warm detection threshold, CPT  =  cold pain threshold, HPT  =  heat pain threshold, MPT  =  mechanical pain threshold, WUR  =  wind-up ratio, PPT  =  pressure pain threshold. * Data were log-transformed before analysis.

## Discussion

The main results of our study are that after exposure to an acute social stressor both groups of participants developed an allodynic effect on thermal stimuli (CPT, HPT), healthy subjects with regard to heat and patients with FMS according to cold stimuli. For both groups a statistical trend towards allodynia/hyperalgesia could be found in the other thermal stimulation. Patients with FMS showed, in addition, stress-related allodynia of PPT.

### Stress-induced thermal allodynia/hyperalgesia

Contrary to our expectations, stress had no analgesic effect in healthy subjects but induced thermal allodynia/hyperalgesia as in patients with FMS. One reason for these results might be an imbalance between central pain-inhibitory effect of stress and stress-induced sensitization of cutaneous nerve fibers [29], [30] leading to thermal allodynia [31], [32]. It is well known that stress induces local expression of cytokines [33]–[35]. Especially the pro-inflammatory cytokines like NGF, IL-6, IL-1, IL-12, IL-18, TNF-a [36]–[40] are able to exert a sensitization effect on cutaneous neural fibers. Our results do not provide insights into a possible contribution of peripheral or central effects of stress-induced cytokine expression. The wind-up ratio normally represents the perceptual correlate of temporal pain summation due to frequency- dependent increase in excitability of spinal dorsal horn neurons [41]. The fact that in neither group a stress-induced increase of the wind-up ratio was detectable suggests a peripheral sensitization effect of cytokines on nociceptors or peripheral opioid receptors [42] in thermal stress-induced allodynia rather than central plasticity changes in the spinal cord.

Another explanation of stress-induced thermal allodynia might be increased anxiety during stress-induction. Under anxiety the experimental procedure might be experienced as more threatening with attention focused upon the stimuli which results in a lowering of the participants’ pain threshold in general [43]. Such an anxiety or attention-related allodynic effect should have been more obvious in our patients with FMS because they showed increased anxiety after stress induction, and patients are known to show increased levels of negative cognitions as catastrophizing [44], [45], which correlate with their level of pain-related impairments [45], [46]. If anxiety had triggered such an effect, it should have affected the thermal and all other stimuli as well. Because the other sensory thresholds as WUR and MPT did not change in face of the stress induction, an anxiety-related effect on thermal allodynia can be ruled out.

No stress-related effect could be detected on pinprick stimulation (MPT) in either group. Possibly different central and peripheral transmission mechanisms are responsible for this phenomenon because nociceptive C-fibers in contrast to a-fibers are under the influence of central descending pain inhibition [47], [48]. Our results suggest an alteration of the processing of mechanical stimulation in our patients with FMS since they did display an increased sensitivity to mechanical stimulation at the baseline testing. That a stress-related sensitization could not be detected in our patients might be due to the low detecting power of the QST in subjects who are already sensitized and already feel pain under conditions of low intensity mechanical stimulation.

### Specific stress-related allodynia/hyperalgesia in patients with FMS

In line with the existing literature, patients with FMS displayed hypersensitivity to heat, cold, mechanical and pressure stimuli at the baseline testing [49]–[51] supporting the hypothesis of an alteration of the central pain processing mechanisms [52]–[54]. Even though our exploratory study design and the limited number of participants preclude a direct comparison of the two groups, the FMS-specific result of a stress-related allodynia/hyperalgesia in response to pressure stimuli might give new insights into the underlying etiological factors in FMS because there might be differences in nociceptive processing originating in cutaneous as compared to deep muscular structures [55]. Both kinds of pain (deep versus cutaneous stimuli) and their underlying spinal and cerebral pathways may be differentially influenced by stress [56].

The syndrome-specific pain in deep tissues might be caused by an increased muscle tone related to the chronic impact of stress [57] resulting in exhaustion and pain in the involved muscles [58]. Such a pain-triggering effect of stress has previously been reported in patients with chronic back pain [59]. This finding may explain the higher prevalence of FMS in females. Stress and tissue damage-related inflammation of the muscle cause comparable alterations in nociceptor sensitivity leading to chronic hyperalgesia and allodynia [60], [61].

Studies in animals have described a chronic-stress-related sensitization of muscle nociceptors that may relate to stress-related cytokine release [62]. Furthermore, increased prostaglandin E2 expression in stressed new-born rats causes hyperalgesia in these animals throughout their lifespan [63], [64]. Since patients with FMS display a correlation of muscle pain with levels of prostaglandin E2 [65] and report increased stress burden in their youth [66], hyperalgesic/allodynic reactions could be based on stress-related alterations of their muscle nociceptors. This underlying pathophysiological mechanism could be exacerbated by an additional acute stressor and explain our finding of stress-related pressure allodynia. Because of the age difference between our group of patients with FMS and healthy subjects, we cannot rule out that the stress-related effect on the pressure pain threshold simply reflects a physiological effect of age. This interesting hypothesis should be tested in future studies of stress-induced effects on pain processing.

### Limitations

We showed stress-induced allodynia in healthy controls and in patients with FMS under quantitative sensory testing in the same area of the subjects’ forearm. To rule out an unlikely but possible local inflammation due to the repetitive stimulation, future studies should apply the stimuli at alternating sites and include a group of subjects who are not under acute stress. This could control for other confounding factors like attention or degree of muscle tension.

Another potential limitation of this study is the fact that we included female participants only. Due to the dominant female distribution in patients with fibromyalgia, the study was conducted exclusively with female participants. Therefore, our results cannot be extrapolated to cover male subjects. Whether stress-induced allodynia/hyperalgesia shows a different pattern in male healthy subjects and patients needs to be tested in future studies.

Furthermore, our study groups are small, which is no doubt a limitation of this study. However, strong effect sizes (η^2^>0.3) of changes in the thermal and pressure pain thresholds were observed, which warrant further studies on mediators of stress.

## Conclusions

Experimental stress has been reported to induce increased pain sensitivity in animals. Our results provide evidence for enhanced pain sensitivity resulting from exposure to experimentally induced stress in humans and suggest differential mechanisms underlying this phenomenon in healthy subjects and patients suffering from a chronic pain condition. Further studies should help reveal the impact of stress and its mediating factors (e.g. cytokines, HPA-axis) on pain sensitivity in chronic pain conditions.
